# Polymers as Stabilizing Excipients for Spray-Dried Protein Formulations

**DOI:** 10.1007/s11095-025-03996-z

**Published:** 2025-12-23

**Authors:** Chanakya D. Patil, Yijing Huang, Kinnari Santosh Arte, Navin Kafle, Harshil K. Renawala, Jiaying Liu, Haichen Nie, Qi Tony Zhou, Li Lily Qu

**Affiliations:** 1 Department of Industrial and Molecular Pharmaceutics, College of Pharmacy, Purdue University, West Lafayette, IN 47907 USA; 2 Merck & Co., Inc., Rahway, NJ 07065 USA

**Keywords:** aggregation, polymer, protein formulations, reconstitution, spray drying

## Abstract

**Purpose:**

Drying is widely used to enhance the storage stability of biologic drug products which are susceptible to degradation in aqueous solutions. Compared to conventional freeze-drying, spray drying offers continuous, high-throughput manufacturing. Stabilizing excipients are critical for protecting proteins from stresses during drying and storage. This study evaluated the potential of polysaccharide- and protein-lysate–based polymeric excipients as alternatives to commonly used stabilizers such as trehalose and mannitol, using bovine serum albumin (BSA) as a model protein.

**Methods:**

Spray-dried BSA formulations were prepared with (2-hydroxypropyl)-β-cyclodextrin (HPβCD), hydrolyzed gelatin, dextran 20 kDa, or sodium carboxymethyl cellulose (NaCMC) polymers, either alone or in combination with trehalose or mannitol. Protein stability was assessed by monitoring monomer loss under stressed storage (40°C, 3 months). Crystallinity and changes in the secondary structure were analyzed using powder X-ray diffraction (PXRD) and solid-state Fourier transform infrared spectroscopy (ssFTIR), respectively. Particle size and size distribution, surface morphology and reconstitution time were also evaluated.

**Results:**

Spray-dried BSA formulations containing HPβCD or hydrolyzed gelatin, either alone or with sugars, exhibited lower monomer loss than the trehalose- or mannitol-only formulations. In contrast, formulations with Dextran 20 kDa and NaCMC showed poor stability. PXRD revealed progressive sodium chloride crystallization during storage. ssFTIR detected secondary structure changes in the BSA over 3 months. The spray-dried powders with polysaccharides generally showed longer reconstitution times than those with polymers.

**Conclusion:**

HPβCD and hydrolyzed gelatin improved the physical stability of spray-dried BSA compared to sugar excipients, which highlights their potential use as stabilizing additives.

**Supplementary Information:**

The online version contains supplementary material available at 10.1007/s11095-025-03996-z.

## Introduction

From 2019 to 2024, approximately 20–30% of drugs approved by the United States Food and Drug Administration (USFDA) were biologics [1], the majority of which were protein drug products [2]. Proteins are susceptible to physicochemical degradation, which reduces efficacy and may trigger immunogenic responses in patients [3]. Removal of water (or dehydration) from protein formulations can slow their degradation and improve shelf stability [4, 5]. Lyophilization or freeze-drying is the most commonly used technique for manufacturing solid-state biologics [6]. However, a key limitation of lyophilization is that it’s a batch-manufacturing process, which often requires several days to achieve an optimally dried product. In contrast, spray drying can be fast and is amenable to continuous, high-throughput and scalable manufacturing, making it a promising alternative to conventional freeze-drying [7]. In recent years, spray-drying has been used for commercial manufacturing of marketed biologic drug products, notably Raplixa® and ExuberaVR® (now discontinued) [8].

Process-related stresses during spray-drying, including thermal, shear and interfacial stress, can lead to protein degradation. Excipients such as sucrose, trehalose, and mannitol, etc., are often used to protect proteins against these stresses [9, 10]. These excipients stabilize proteins in the solid-state through two proposed mechanisms. The vitrification theory suggests that sugars form a glassy matrix, reducing molecular mobility (α- and β-relaxations) and improves storage [11, 12]. Alternatively, the water replacement theory proposes that the loss of hydrogen bonds between protein and water on dehydration are replaced by those between protein and sugar molecules, contributing to their conformational and colloidal stability during and after the drying process and on reconstitution in aqueous solution [11, 12]. Sugar alcohols like mannitol and sorbitol have also proven to be effective in stabilizing proteins during spray-drying [13–15]. Raplixa® and ExuberaVR® contain trehalose and mannitol as stabilizers, respectively [16, 17].

In addition to the commonly used disaccharides (sucrose, trehalose), oligosaccharides such as cyclodextrins (CDs) have gained interest as stabilizing excipients in protein formulations. Among them, (2-hydroxypropyl)-β-cyclodextrin (HPβCD) has been evaluated owing to its unique structural properties. HPβCD possesses a hydrophobic core, which can interact with hydrophobic protein residues and influence stability [18]. HPβCD is also thought to exhibit some surface activity and minimize protein aggregation at the air–water interface [19, 20]. Furthermore, HPβCD can contribute to protein stabilization during drying by forming hydrogen bonds with proteins to influence conformational stability and reduce aggregation, highlighting its utility as a multifunctional excipient with surfactant-like and protective properties [21–23]. HPβCD has been reported as a stabilizer for spray-dried protein formulations of humanized immunoglobulin G (IgG) antibody and showed comparable stability as trehalose [24, 25].

Polymers can be used as stabilizers because of their matrix-forming capability in the solid state [26, 27]. They generally exhibit a high glass transition temperature (> 100°C), which correlates to lower molecular mobility at the ambient storage temperatures [28, 29]. Carbohydrate-based polymers, such as dextran and sodium carboxymethyl cellulose (NaCMC), have been explored for their potential in protein stabilization [30]. Dextran has been investigated as stabilizing and matrix forming excipient in some spray-dried protein formulations; however, in comparison to trehalose, it has generally shown poor ability to form stabilizing interactions with proteins, perhaps due to their large molecular weights and tendency to phase separate from proteins in a solid matrix [31, 32]. Interestingly, in a recent study, Nguyen et al. showed that BSA in spray-dried formulations containing high molecular weight dextran (~ 2000 kDa) was more stable against aggregation compared to those with comparatively low molecular weight dextran (6 and 70 kDa), which was postulated to be due to better miscibility of BSA monomers within polymer coils of 2000 kDa dextran [32]. However, in this study, BSA formulations containing dextran (6, 70 and 2000 kDa) showed overall poor aggregation stability than those containing trehalose. Other reports have suggested that dextran may contribute to protein stability against interfacial stresses during spray-drying by reducing adsorption at the air–water interface [33–35]. While NaCMC has not been extensively studied as a protein stabilizer, some reports indicate its use in protein formulations to protect against stresses associated with spray-drying [30, 36].

Gelatin has been used in biologic formulations, including a few USFDA-approved vaccines [37]. It’s hydrolyzed form has been shown to protect viruses and viral proteins from stresses experienced during freeze–thaw cycles and drying [38]. However, the mechanism by which gelatin stabilizes viruses and proteins is not fully understood. Hydrolyzed gelatin is an enzymatically cleaved product of gelatin, which improves its aqueous solubility at ambient temperature. It’s not a commonly used excipient in protein formulations but is found in some lyophilized vaccine products such as MMRII® and Varivax® [38–41]. As gelatin and its byproducts are derived from animal collagen, there can be raw material variability from different sources of gelatin, which may be among the reasons for its infrequent use in parenteral drug products.

In the present study, BSA was formulated and spray-dried with different polysaccharide ((2-hydroxypropyl)-β-cyclodextrin, sodium carboxymethyl cellulose, dextran) and protein-lysate (hydrolyzed gelatin) excipients, either alone or in combination with trehalose (a disaccharide) and mannitol (a sugar alcohol). These excipients represent a range of chemical compositions, molecular weights, and structural features that could influence protein stabilization and particle characteristics during spray drying and on storage. HPβCD, a cyclic oligosaccharide with a hydrophobic cavity, was included for its reported surfactant-like and hydrogen-bonding capabilities; hydrolyzed gelatin, a protein-lysate, was selected for its protective properties in lyophilized viral vaccines; NaCMC was included as a charged, linear polysaccharide that can form an amorphous matrix; and dextran (20 kDa) was chosen as a representative neutral, branched polysaccharide with relatively high molecular weight. Aggregation stability of spray-dried BSA exposed to stressed conditions (40°C, 3 months) was evaluated by size exclusion chromatography. Secondary structural changes were monitored by solid-state Fourier transform infrared spectroscopy and correlated to monomer loss on aggregation. Powder X-ray diffraction provided insights into excipient crystallization on spray-drying and storage. Particle surface morphology was observed using scanning electron microscopy for the different BSA-polymer spray-dried powders. Overall, this study aims to evaluate the effectiveness of polymers in stabilizing spray dried proteins, either alone or when used together with commonly used excipients, trehalose and mannitol, and assess their particle characteristics.

## Materials and Methods

### Materials

Bovine serum albumin (BSA) (Purity ≥ 98%), gelatin hydrolysate enzymatic (hydrolyzed gelatin), (2-hydroxypropyl)-β-cyclodextrin (HPβCD), trehalose dihydrate, D-mannitol, sodium citrate dihydrate, citric acid, and polysorbate 80 were procured from Millipore Sigma (St. Louis, MO, USA). Dextran (Avg. MW 20,000 Da) was procured from Alfa Aesar (Ward Hill, MA, USA). Carboxymethyl cellulose sodium (NaCMC) (low viscosity) was procured from Spectrum Chemical (New Brunswick, NJ, USA). Sodium chloride was procured from Avantor (Radnor Township, PA, USA). The spray-dried formulations were stored in 20R type I borosilicate glass vials stoppered with 20 mm two-legged chlorobutyl stoppers and sealed with 20 mm aluminum crimps. For stability studies, samples were filled in 2R type I borosilicate glass vials with 13 mm two-legged chlorobutyl stoppers, and 13 mm aluminum crimp seals.

### Formulation Preparation

All the formulations contain bovine serum albumin (BSA), sodium citrate buffer (5.16 mg/mL), sodium chloride (8.77 mg/mL), and polysorbate 80 (0.25 mg/mL). Except the control formulation, all the other formulations were prepared using 1:1 or 1:2 (w/w) ratio of BSA to polymers/trehalose/mannitol. The detailed formulation composition is reported in Table I. 20 mM sodium citrate buffer was prepared at pH 6.0. BSA was dissolved in the citrate buffer and dialyzed using a dialysis cassette (Slide-A-Lyzer, 10,000 MWCO, Thermo Scientific, Rockford, IL). Dialysis was performed to reduce the low molecular weight impurities present in the vendor BSA. Polymer/sugar solutions in citrate buffer were added to the dialyzed BSA solution to reach the final protein and excipient concentrations listed in Table I. The total solid content was 2.44% w/v for the control BSA formulation (C) without a polymer or sugar excipient, 3.44% w/v for formulations with single polymeric or sugar excipients (H, N, G, D, T, M), and 4.4% w/v for formulations with two polymeric or sugar excipients (HT, NT, GT, DT, HM, NM, GM, DM).

**Table I:** Composition of spray-dried BSA formulations

#	Formulations	BSA (mg/mL)	Stabilizing excipients		Total solid content (mg/mL)
			Excipient 1 (10 mg/mL)	Excipient 2 (10 mg/mL)	
0	Control (C)	10.0	–	–	24.4
1	HPβCD (H)		HPβCD		34.4
2	NaCMC (N)		NaCMC		
3	Hydrolyzed Gelatin (G)		Hydrolyzed Gelatin		
4	Dextran 20 kDa (D)		Dextran 20 kDa		
5	Trehalose (T)		Trehalose		
6	Mannitol (M)		Mannitol		
7	HT		HPβCD	Trehalose	44.4
8	NT		NaCMC		
9	GT		Hydrolyzed Gelatin		
10	DT		Dextran 20 kDa		
11	HM		HPβCD	Mannitol	
12	NM		NaCMC		
13	GM		Hydrolyzed Gelatin		
14	DM		Dextran 20 kDa		

### Spray-Drying Process

The aqueous BSA formulations listed in Table I were spray-dried using Büchi Mini Spray Dryer B-290 (New Castle, DE, USA). The process parameters include an inlet temperature of 120°C and the outlet temperature ranging from 60–65°C, according to previous studies [42–44]. The liquid feed rate was set at 2 mL/min with an atomization air rate of 600 L/h [14]. The spray-dried powders were collected in 20 mL (20R) borosilicate vials, stoppered and crimped for further characterization. The percentage yield was calculated using the ratio of the actual yield to the theoretical yield for each formulation.

### Stressed Storage Stability

Stability testing was conducted under stressed storage conditions in a temperature-controlled chamber maintained at 40°C. Spray-dried powders were filled into 2 mL (2R) Type I borosilicate glass vials within a humidity-controlled environment (glove box purged with dry nitrogen gas and maintained at around 20% relative humidity). The vials were subsequently stoppered, sealed, and crimped to minimize moisture ingress. The sealed vials were then stored at 40 °C under ambient humidity conditions for a duration of 90 days (3 months).

### Karl Fischer (KF) Titration

The residual moisture content in the spray-dried formulations was measured using a 917 KF Coulometer (Metrohm, Riverview, FL, USA). Approximately 2–3 mg spray-dried powder for each formulation was weighed in triplicate. 1 mL of anhydrous methanol (DriSolv®, Millipore Sigma St. Louis, MO, USA) was added to each sample and the mixture was injected into the Coulomat reagent (Honeywell Research Chemicals, Seelze, Germany) in the titration vessel of the instrument. Moisture content was recorded in parts per million (ppm) and calculated in % w/w using the weights of the powder and the mixture injected.

### Particle Size Distribution

The particle size of spray-dried formulations was analyzed using Mastersizer 3000 (Malvern Panalytical, Malvern, UK), equipped with an Aero S unit. The feed rate was set at 60% while compressed air (4 bar) was used to disperse the particles inside the Aero S chamber. Approximately 60 mg of powder was used to perform analyses in triplicate. Percentile distribution at 10% (D_10_), 50% (D_50_) and 90% (D_90_) and span was calculated using the instrument software.

### Particle Morphology

Scanning electron microscopy (NOVA nanoSEM, FEI Company, Hillsboro, OR, USA) was used to visualize the particle morphology of the spray-dried formulations. The dried powder for each formulation was gently placed on a carbon tape attached to the sample holder, and each sample was sputter coated with platinum for 60 s. The samples were analyzed with a beam voltage of 5 kV and a spot size of 2.5.

### Powder X-Ray Diffraction (PXRD)

Rigaku SmartLab X-ray diffractometer (The Woodlands, TX, USA) was used to analyze crystallinity in the spray-dried formulations. The system was equipped with a Cu Kα X-ray source. The samples were gently placed over the glass sample holder and diffractogram was collected as 2θ ranging from 4° to 40° with a step size of 0.02°. Current was set at 44 mA with a voltage of 40 kV.

### Solid-State Fourier Transform Infrared Spectroscopy (ssFTIR)

Changes in the secondary structure of BSA were monitored using Thermo Nicolet Nexus FTIR (Thermo Scientific, Waltham, MA, USA). The instrument was equipped with a Smart iTR accessory. 128 scans of the absorbance spectra were obtained from 800 cm^−1^ to 4000 cm^−1^. Analyses were performed in attenuated total reflectance mode at a resolution of 4 cm^−1^.

### Size Exclusion Chromatography (SEC)

Monomer content and high molecular weight species (aggregates) were quantified using SEC. The stability samples were stored at 40°C for 3 months and analyzed at 0, 30, 60, and 90-day intervals. The percentage area of the monomer peak to the aggregate peak was determined using five replicates of each formulation at all time points. The dried samples were reconstituted to obtain a concentration of 1 mg/mL BSA using deionized water. 0.1 M sodium phosphate buffer (pH 6.8) was used as the mobile phase. TOSOH TSKgel® G3000SWXL column (7.8 mm I.D. × 30 cm, 5 µm) was connected to a 1260 Infinity II series high-performance liquid chromatography (HPLC) system (Agilent Technologies, Santa Clara, CA, USA). The isocratic flow rate was set to 1 mL/min. BSA monomer content was analyzed at 280 nm using photodiode array (PDA) detector (Agilent, Waldbronn, Germany). The total percentage monomer loss after 90 days (T_90_) was calculated by subtracting the % monomer content at T_90_ from the monomer content at T_0_.

### Reconstitution Time

Appropriate amounts (24.2–44.2 mg) of spray-dried solids were weighed to obtain a reconstitute volume of 1 mL (calculated using total solid content). BSA concentration post-reconstitution was targeted at 10 mg/mL for all formulations. The spray-dried solids were weighed in 2R type I borosilicate glass vials, 1 mL of water for injection (WFI) was added to the powders. The vials were gently swirled once and placed on a flat surface. Reconstitution time was recorded as the time needed for the particles to dissolve and form a clear solution, as observed by visual inspection.

### Statistical Analysis

One-way ANOVA with Tukey’s multiple comparison test was performed post-hoc using GraphPad Prism software version 9 (GraphPad, La Jolla, CA, USA), and p-values were determined. (**** stands for *p* < 0.0001, *** for *p* < 0.001, ** for *p* < 0.01 and * for *p* < 0.05).

## Results

### Residual Moisture Content and Percentage Yield

Moisture content of the spray-dried samples at time 0 (T_0_) was determined to be in the range of 2–3% w/w. It increased slightly to 3–5% w/w over 3-months storage (Table II). The percentage yield on spray-drying ranged from 64–84%.

**Table II:** Residual moisture content (*n* = 3; Mean ± SD), percentage yield, and particle size distribution (*n* = 3; Mean ± SD)

#	Formulations	% Moisture Content (w/w)		% Yield	Particle size distribution			
		T_0_	T_90_		D_10_ (µm)	D_50_ (µm)	D_90_ (µm)	Span
0	Control (C)	2.8 ± 0.3	3.9 ± 0.3	73.7	0.3 ± 0.0	2.5 ± 0.2	5.6 ± 0.3	2.2 ± 0.0
1	HPβCD (H)	2.7 ± 0.3	4.3 ± 0.2	75.2	0.3 ± 0.0	2.0 ± 0.0	4.6 ± 0.2	2.1 ± 0.1
2	NaCMC (N)	3.1 ± 0.5	3.6 ± 0.3	78.7	0.3 ± 0.0	2.3 ± 0.0	6.2 ± 0.5	2.5 ± 0.2
3	Hydrolyzed Gelatin (G)	2.8 ± 0.6	3.7 ± 0.7	65.4	0.3 ± 0.0	2.3 ± 0.1	5.0 ± 0.0	2.1 ± 0.0
4	Dextran 20 k (D)	2.4 ± 0.2	4.3 ± 0.4	80.4	0.3 ± 0.0	2.5 ± 0.0	5.4 ± 0.0	2.1 ± 0.0
5	Trehalose (T)	2.5 ± 0.4	3.8 ± 0.4	64.0	0.3 ± 0.0	2.6 ± 0.1	6.0 ± 0.2	2.2 ± 0.0
6	Mannitol (M)	2.2 ± 0.4	3.2 ± 0.8	73.7	0.3 ± 0.0	2.2 ± 0.1	5.2 ± 0.1	2.2 ± 0.1
7	HT	2.9 ± 0.6	4.8 ± 0.5	81.5	0.3 ± 0.0	2.4 ± 0.0	6.4 ± 0.1	2.6 ± 0.0
8	NT	3.3 ± 0.2	4.4 ± 0.1	81.4	0.3 ± 0.0	2.6 ± 0.1	6.0 ± 0.1	2.2 ± 0.0
9	GT	3.3 ± 0.4	4.1 ± 0.1	81.7	0.3 ± 0.0	2.4 ± 0.1	5.3 ± 0.3	2.1 ± 0.1
10	DT	2.4 ± 0.4	3.1 ± 0.5	76.6	0.3 ± 0.0	2.5 ± 0.0	5.5 ± 0.1	2.0 ± 0.0
11	HM	2.1 ± 0.3	4.5 ± 0.3	80.8	0.3 ± 0.0	2.4 ± 0.2	5.3 ± 0.4	2.1 ± 0.1
12	NM	2.5 ± 0.4	2.8 ± 0.4	84.3	0.3 ± 0.0	2.6 ± 0.1	6.0 ± 0.1	2.5 ± 0.4
13	GM	2.2 ± 0.1	3.4 ± 0.8	82.7	0.3 ± 0.0	2.6 ± 0.0	6.9 ± 0.6	2.6 ± 0.2
14	DM	2.1 ± 0.3	2.9 ± 0.2	76.6	0.3 ± 0.0	2.5 ± 0.0	5.4 ± 0.1	2.1 ± 0.0

### Particle Size Distribution

The median particle diameter (D_50_) was 2.0–3.0 µm, while the D_10_ and D_90_ were observed to be ~ 0.3 µm and 4.6–6.9 µm, respectively. The span was 2.0–2.6. The comparable D_50_ and span values across formulations indicated that both the median particle diameter and the overall particle size distribution were not significantly influenced by the type of polymer or sugar excipients at a given total solid content. Table II provides details of the particle size distribution for all formulations.

### Particle Morphology

**Fig. 1:**
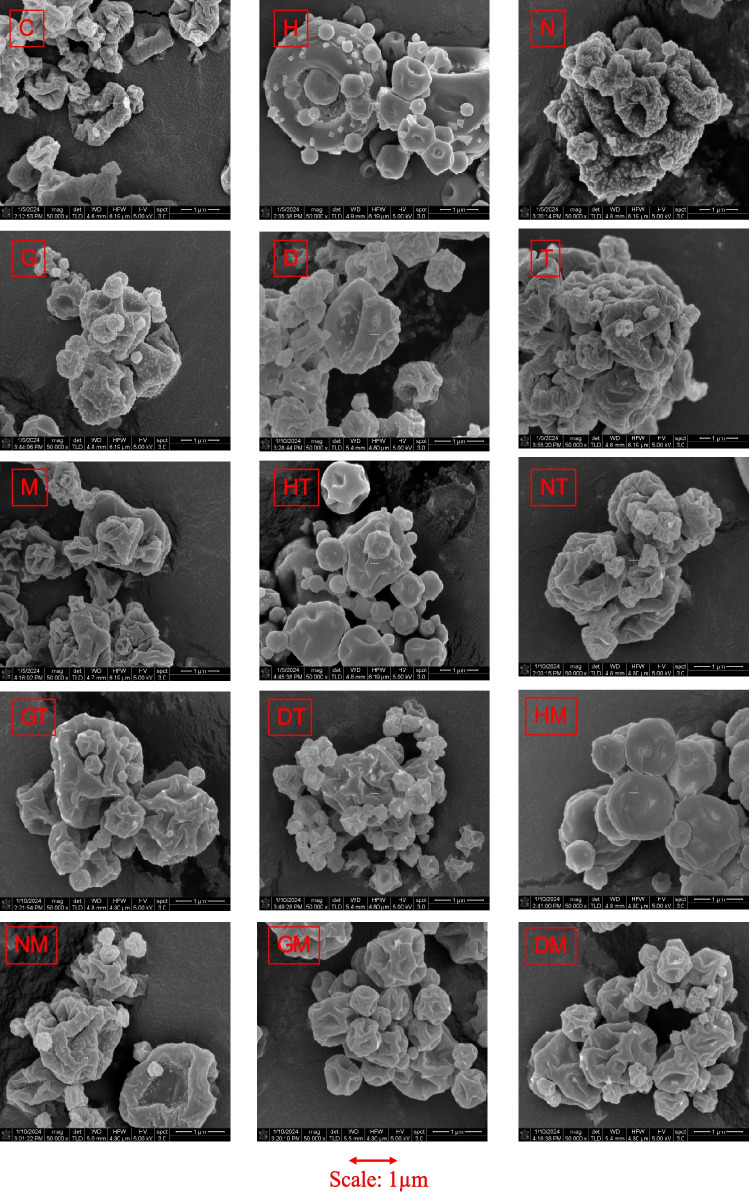
Morphology of spray-dried particles at time 0.

Spray-dried particles for all formulations were observed to have tiny cube-like or irregular deposits on their surface (Fig. 1). HPβCD-containing formulations (formulations H, HT, and HM) were observed to have fewer corrugations and a smoother surface compared to formulations containing other polymeric excipients. Combining HPβCD with trehalose or mannitol also resulted in smoother particle surface than formulations with a combination of these sugars with other polymers. All formulations, except H, HT, and HM, were found to have collapsed particle morphology. No significant changes in particle morphology were observed after 3-months storage at 40°C (Figure S1).

### Size Exclusion Chromatography (SEC)

**Fig. 2:**
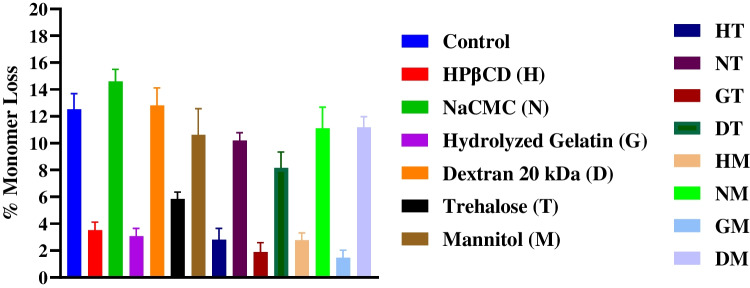
Monomer loss of BSA spray-dried formulations over 3 months at 40 °C (*n* = 5; Mean ± SD).

BSA formulations containing a single polymeric or sugar excipient – HPβCD (H), hydrolyzed gelatin (G), or trehalose (T) – showed significantly lower monomer loss than those containing NaCMC (N), dextran 20 kDa (D) or mannitol (M) over 3 months storage at 40°C. Formulations with trehalose in combination with polysaccharide (HT, NT, DT) or protein-lysate (GT) excipients showed lower monomer loss and better stabilization of BSA compared to their corresponding formulations with single polymeric excipient (H, N, D, G). Although, mannitol containing formulation (M) showed poor stability of BSA (~ 10% monomer loss); formulations with mannitol combined with HPβCD (HM) or hydrolyzed gelatin (GM) showed better stabilization of BSA with ~ 3.0% and ~ 1.5% monomer loss, respectively, over 3-months storage at 40°C. Mannitol containing formulations with polysaccharides such as dextran 20 kDa (DM) and NaCMC (NM) showed slightly lower BSA monomer loss than those with polysaccharide alone (D, N). Overall, the physical stability of BSA was significantly improved in formulations containing a combination of HPβCD or hydrolyzed gelatin with trehalose (HT, GT) or mannitol (HM, GM) (Fig. 2). The statistical comparisons along with their *p*-values are listed in Table S1 in Supplementary Information.

### Powder X-Ray Diffraction (PXRD)

**Fig. 3:**
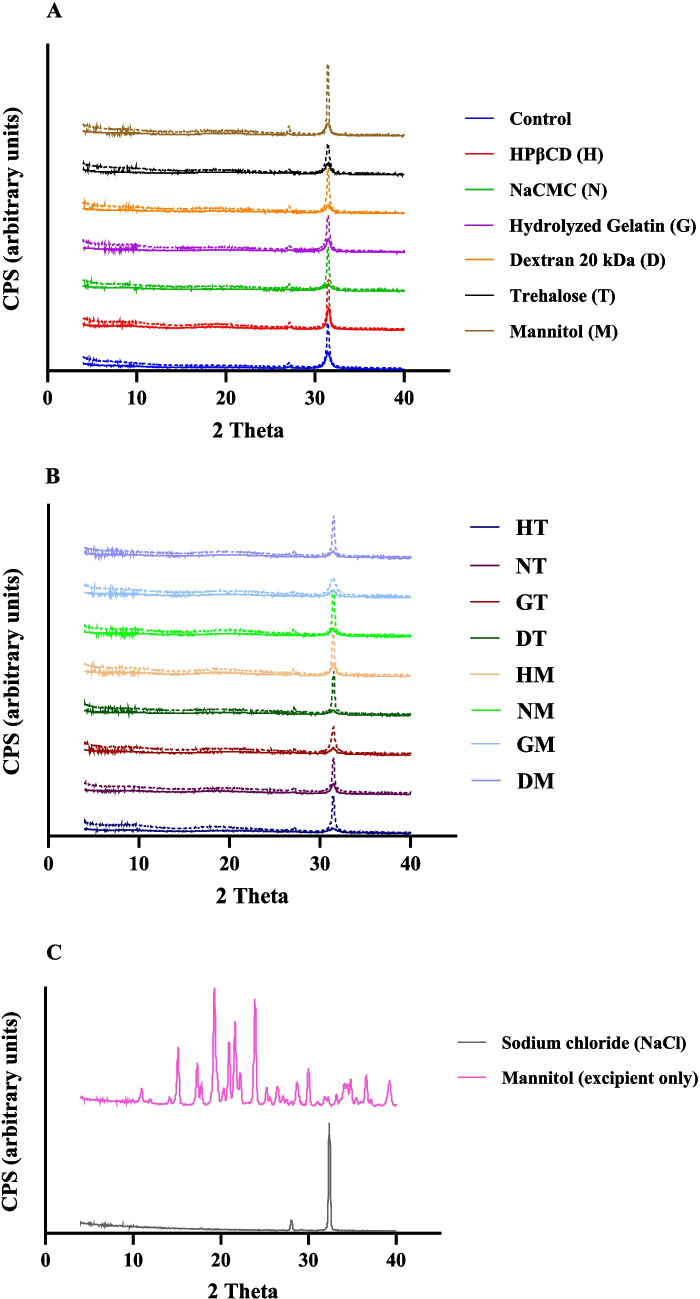
X-ray diffractograms of spray-dried BSA-polymer formulations stored at 40°C for 90 days; **A**: control & single polymer/sugar formulations; **B**: combination formulations of polymers with trehalose/mannitol; **C**: spray-dried excipients only. Solid line represents time 0 (T_0_) samples; dashed line represents samples at 90 days (T_90_) of stressed storage.

The X-ray diffractograms were obtained at time 0 (T_0_) and after 3 months of storage (T_90_) at 40°C (Fig. 3A, B). The crystallization peaks from individual excipients were obtained for spray-dried sodium chloride and mannitol (Fig. 3C). All spray-dried formulations showed presence of sharp peaks at 2θ ~ 27° and 32° at T_0_, which can be attributed to crystallization of sodium chloride (NaCl). The peak intensity for crystalline NaCl increased over 3-months storage at elevated temperature for all formulations (Fig. 3, Table S2). Among the formulations studied, those containing hydrolyzed gelatin and trehalose (GT) or mannitol (GM) demonstrated the lowest NaCl peak intensities at day 90 (Fig. 3B), whereas the mannitol-only formulation (M) exhibited the highest NaCl peak intensity (Fig. 3A). None of the mannitol-containing formulations (M, HM, NM, GM, DM) showed peaks corresponding to crystalline mannitol, indicating that it remained amorphous over 3-month storage.

### Solid-State Fourier Transform Infrared Spectroscopy (ssFTIR)

**Fig. 4:**
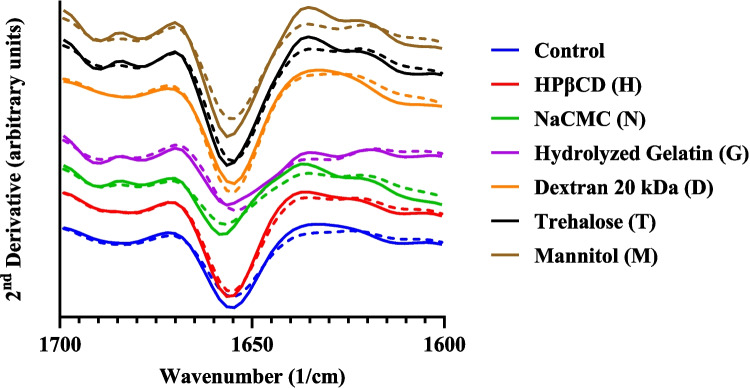
The 2^nd^ derivative ssFTIR spectra of spray-dried BSA formulations. Solid line represents time 0 (T_0_) samples; dashed line represents samples at 90 days of stressed storage (T_90_).

The 2nd derivative ssFTIR analysis is consistent with the presence of α-helix from the Amide I signal (∼ 1656 cm^−1^) of BSA (Fig. 4). The control formulation and those containing NaCMC (N), hydrolyzed gelatin (G), and mannitol (M) showed changes in the secondary structure of BSA after 3-months storage at elevated temperature (40°C). No major changes in the secondary structure of BSA were observed for formulations containing HPβCD (H), dextran 20 kDa (D), and trehalose (T).

### Reconstitution Time

**Fig. 5:**
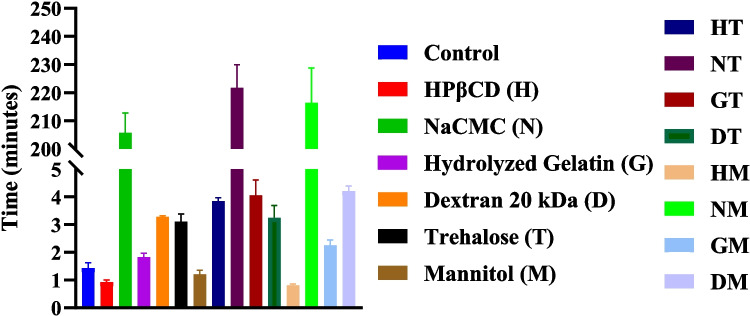
Reconstitution time for the spray-dried formulations (*n* = 3; Mean ± SD).

Spray-dried BSA formulations containing HPβCD or mannitol alone or in combination (H, M, HM) were observed to have the shortest reconstitution time (~ 1 min or less) among all, whereas NaCMC containing formulations (N, NT, NM) showed the longest reconstitution time (> 30 min) (Fig. 5). BSA formulations containing gelatin (G), dextran 20 kDa (D), trehalose (T) and their combinations (GT, DT) dissolved in < 5 min.

## Discussion

Spray drying has been increasingly reported as an effective drying technique for biologics including small peptides or proteins, antibodies and gene therapy [45–48], and has emerged as a promising alternative to conventional freeze-drying process. Excipients such as trehalose, sucrose and mannitol are often used as stabilizers for freeze-dried and spray-dried proteins to protect against stresses involved in these processes. Stresses during freeze-drying involve cryo-concentration, cold-denaturation and presence of ice/water interface, whereas stresses during spray-drying involve exposure to elevated temperatures, shear from atomization and presence of air/water interface [49]. Both drying techniques involve removal of water (dehydration) and the resulting loss of stabilizing hydrogen-bonded interactions with proteins, which are replaced by interactions with sugar excipients [12]. In addition to commonly used disaccharide excipients such as trehalose and sucrose, natural (complex carbohydrates, protein lysates) or synthetic polymers (e.g., polyvinylpyrrolidone) can also be used to stabilize proteins in the solid-state and confer desirable product/particle characteristics to freeze- and spray-dried formulations. The objective of this study was to evaluate the stabilization potential of polymeric carbohydrates of increasing complexity, including mannitol (sugar alcohol), trehalose (disaccharide), 2-hydroxypropyl-β-cyclodextrin (HPβCD, oligosaccharide), dextran 20 kDa and sodium carboxymethylcellulose (polysaccharides), and hydrolyzed gelatin (protein lysate) in spray-dried bovine serum albumin (BSA) formulations and assess their particle characteristics.

Particle size of spray-dried powders can be correlated to the size of droplets formed during the atomization step and the total solid content of formulations [50]. In our study, even though the total solid content across formulations was different (ranging from 2.4% to 4.4% w/v), the spray-dried powders showed similar particle size and size distribution for the different polymeric excipients used (Table II). Particle morphology in spray-dried solids is influenced by several factors, including formulation composition, drying kinetics and process parameters [51, 52]. Among the spray-dried formulations studied, those containing HPβCD showed relatively smooth surface with less corrugations, even when combined with trehalose or mannitol, compared to other formulations (Fig. 1). Components with surface activity can enrich on particle surface and modify particle morphology during spray drying [53, 54]. HPβCD has been reported to have surface activity [19], which may have influenced particle morphology. All spray-dried formulations showed irregular protrusions on their particle surfaces, which may have been from crystallization and surface deposition of sodium chloride (NaCl), as detected by PXRD (Fig. 3). Similar surface morphologies have been reported in previous studies of spray-dried formulations containing NaCl, which have been attributed to its crystallization [55–57].

Spray-dried BSA formulations were stored under stressed conditions (40°C, 3 months) to evaluate the stabilizing potential of different polymeric excipients. Aggregation studies by size exclusion chromatography (SEC) showed that spray-dried formulations containing HPβCD and hydrolyzed gelatin, as single polymeric excipient, had better storage stability of BSA compared to formulations with single sugar excipient, trehalose or mannitol (Fig. 2). On the contrary, BSA formulations containing dextran 20 kDa and NaCMC showed high monomer loss (~ 13–15%) and poor stability comparable to the control formulation without any protective excipients. Long chain high molecular weight polymers, such as dextran, have shown poor ability to form stabilizing interactions with proteins and were found to phase separate in the solid-state, which has been correlated to increased aggregation on long-term storage [32]. While mannitol remained amorphous (Fig. 3), it showed insufficient stabilization and high monomer loss of BSA, whereas formulations containing a combination of HPβCD or hydrolyzed gelatin with mannitol were among the most stable (Fig. 2). HPβCD has been reported to stabilize proteins in aqueous and solid formulations, where the stabilizing effect was hypothesized to be due to interactions of hydrophobic protein residues with the hydrophobic core of HPβCD [58]. In addition, HPβCD may reduce protein degradation at interfaces due to its surface activity [22, 58, 59]. Hydrolyzed gelatin, to our knowledge, has not been previously reported as a stabilizing excipient for spray-dried protein formulations.

Solid-state characterization studies of the spray-dried powders, including residual moisture content by KF titration, crystallinity by PXRD and secondary structure analysis by ssFTIR were performed to further probe the differences in stability profiles observed by SEC. High residual moisture is correlated to poor protein stability in solid formulations from increased water activity and molecular mobility [12]. Furthermore, high residual moisture can lower the glass transition temperature (T_g_) of spray-dried solids, accelerating crystallization and phase separation of protective excipients, thereby reducing stabilizing protein-excipient interactions [60, 61]. In our study, all spray-dried formulations showed an initial residual moisture content of ~ 2–3% (w/w) (Table II). Although there was slight increase in moisture content (to ~ 3–5% w/w) over 3-month storage, it did not correlate to differences in monomer loss by SEC (Fig. 2).

Crystallization of protective excipient results in phase separation in the solid-state, which can reduce stabilizing interactions with proteins and lead to their degradation [62]. Sodium chloride (NaCl) is often included in protein formulations to modulate ionic strength, viscosity and colloidal stability in aqueous solutions [63, 64]. In our study, NaCl recrystallized on spray-drying, and the crystallinity increased over 3-month storage for all formulations (Fig. 3) (Table S2). This could be due to the elevated storage temperature and residual moisture, among other factors, which can increase molecular mobility and accelerate crystallization. Although, NaCl recrystallization can affect BSA stability, it was not correlated to the observed trend in monomer loss by SEC (Table S2, Fig. 2). Moreover, mannitol has been reported to recrystallize at 2:1 w/w ratio (BSA: mannitol) in spray-dried BSA formulations [65]. Previous studies have suggested that NaCl and mannitol can influence each other’s crystallization in frozen solutions, in a manner that was dependent on their weight ratios, solution cooling or heating rates and presence of other components like proteins [66, 67]. In our study, NaCl crystallized but mannitol did not in spray-dried formulations containing roughly equal weight ratios of NaCl (~ 9 mg/mL), mannitol, BSA and polymeric excipients (10 mg/mL each).

Changes in the secondary structure of BSA, as detected by ssFTIR, informed on its conformational stability in the spray-dried formulations. BSA is an alpha-helix rich protein [68]. The ssFTIR spectra for spray-dried BSA formulations containing trehalose and HPβCD were consistent with the presence of helical BSA (Fig. 4), which did not show significant changes over 3 months storage. On the contrary, the spectrum for NaCMC and mannitol containing formulations showed marked changes in the peak intensity and position on storage. These observations were correlated with relatively lower aggregation and better physical stability of trehalose and HPβCD containing formulations compared to those with NaCMC and mannitol (Fig. 2). This was not true for formulations containing hydrolyzed gelatin and dextran 20 kDa (Fig. 2). As hydrolyzed gelatin is a protein excipient, it shows absorbance in the IR spectrum and can interfere with ssFTIR analysis of BSA [69]. In mannitol-containing formulations, secondary structure perturbations were evident despite the absence of detectable crystallization, indicating that amorphous mannitol was still unable to adequately stabilize spray-dried BSA. Interestingly, Chen et al. reported similar destabilization trends for mannitol–BSA spray-dried system (1:1 w/w ratio), although mannitol crystallization during storage was not evaluated in their study [43]. The consistency in stability outcomes suggests that mechanisms other than crystallization, such as poor matrix homogeneity and limited hydrogen bonding with BSA, could be contributing to mannitol’s limited ability to stabilize BSA in spray-dried formulations.

Reconstitution time is an important quality attribute for dried protein formulations, especially for injectable drug products. Interestingly, HPβCD alone and in combination with mannitol showed shortest reconstitution time among all (< 1 min; Fig. 5). Mannitol has been reported to reduce reconstitution time of lyophilized solids by improving wettability and easing the disintegration process [70, 71]. The role of HPβCD in improving the reconstitution time of spray dried solids is unclear and can also be influenced by formulation and process factors. On the contrary, spray-dried formulations containing NaCMC remained undissolved even after 30 min post reconstitution with water, likely due to inherently slow dissolution of the polymer and increased viscosity of its aqueous solution. While the differences in the reconstitution time of the polymer/sugar/protein spray-dried powders provide preliminary indications of their dissolution behavior, the kinetics of drug release from the spray-dried particles in presence of polymers need further investigation.

Overall, HPβCD and hydrolyzed gelatin were better stabilizers against aggregation for spray-dried BSA under stressed conditions (40°C, 3 months). Further studies are needed to understand the mechanism of protein stabilization by these excipients. HPβCD and hydrolyzed gelatin are both FDA-approved excipients for parenteral formulations [38, 72], and can potentially be used as additive excipients complementing the stabilizing effects of trehalose and mannitol in spray-dried protein formulations.

## Conclusion

This comparative study highlights the use of polysaccharide and protein-lysate based excipients as stabilizers for spray-dried protein formulations. Spray-dried BSA formulated with HPβCD or hydrolyzed gelatin in combination with trehalose or mannitol showed lowest aggregation propensity and improved physical stability compared to formulations containing trehalose or mannitol alone under the stressed storage conditions used (40°C, 3 months), suggesting that these excipients can enhance the stability of spray-dried protein formulations. Spray-dried powders containing these excipients showed spherical morphology and rapid reconstitution time. On the contrary, spray-dried BSA formulations containing dextran 20 kDa and NaCMC polymers showed poor aggregation stability and relatively long reconstitution time. Powder X-ray diffraction (PXRD) showed presence of crystalline sodium chloride in all formulations; however, it was not correlated to storage stability of BSA. ssFTIR analysis showed perturbations in the secondary structure of BSA in formulations containing mannitol and NaCMC, which correlated with greater aggregation in these formulations under stressed conditions; however, such correlation was not observed for BSA formulations containing dextran 20 kDa and hydrolyzed gelatin. While HPβCD and hydrolyzed gelatin seem to be promising excipients for protein stabilization in the solid-state, the mechanism of stabilization needs further investigation.

## Supplementary Information

Below is the link to the electronic supplementary material.

ESM 1 (DOCX 3.47 MB)

## Data Availability

The datasets generated in this study are available from the authors on reasonable request.
