# Supplementary material for: Polymers as Stabilizing Excipients for Spray-Dried Protein Formulations
Source: Pharm Res. 2025 Dec 23;43(2):573–85. doi: 10.1007/s11095-025-03996-z (PMC12963100; doi:10.1007/s11095-025-03996-z)
Supplement: Supplementary file 1 — (DOCX 3.47 MB) [file 11095_2025_3996_MOESM1_ESM.docx]

**Supplementary Information**

**Polymers as Stabilizing Excipients for Spray-dried Protein Formulations**

Chanakya D. Patil^1^, Yijing Huang^1^, Kinnari Santosh Arte^1^, Navin Kafle^2^_,_ Harshil K Renawala^2^, Jiaying Liu^2^, Haichen Nie^1^, Qi (Tony) Zhou^1, *^, Li (Lily) Qu^1, *^

^1^ Department of Industrial and Molecular Pharmaceutics, College of Pharmacy, Purdue University, West Lafayette, IN 47907, USA

^2^ Merck & Co., Inc., Rahway, NJ 07065, USA

^*^ Corresponding authors: Qi (Tony) Zhou**,** email: [tonyzhou@purdue.edu](mailto:tonyzhou@purdue.edu); Li (Lily) Qu, email: [qu135@purdue.edu](mailto:qu135@purdue.edu)

**Table S1.** Statistical comparisons of monomer loss in spray-dried BSA formulations containing different polymeric and sugar excipients by size exclusion chromatography (see Figure 2 for % monomer loss data)

| **Tukey's multiple comparisons test** | **Summary** | **Adjusted P Value** |
| --- | --- | --- |
| Control vs. HPBCD (H) | **** | <0.0001 |
| Control vs. NaCMC (N) | ns | 0.1081 |
| Control vs. Hydrolyzed Gelatin (G) | **** | <0.0001 |
| Control vs. Dextran 20 kDa (D) | ns | >0.9999 |
| Control vs. Trehalose (T) | **** | <0.0001 |
| Control vs. Mannitol (M) | ns | 0.1841 |
| Control vs. HT | **** | <0.0001 |
| Control vs. NT | * | 0.0355 |
| Control vs. GT | **** | <0.0001 |
| Control vs. DT | **** | <0.0001 |
| Control vs. HM | **** | <0.0001 |
| Control vs. NM | ns | 0.6462 |
| Control vs. GM | **** | <0.0001 |
| Control vs. DM | ns | 0.7322 |
| HPBCD (H) vs. NaCMC (N) | **** | <0.0001 |
| HPBCD (H) vs. Hydrolyzed Gelatin (G) | ns | >0.9999 |
| HPBCD (H) vs. Dextran 20 kDa (D) | **** | <0.0001 |
| HPBCD (H) vs. Trehalose (T) | * | 0.0354 |
| HPBCD (H) vs. Mannitol (M) | **** | <0.0001 |
| HPBCD (H) vs. HT | ns | 0.9984 |
| HPBCD (H) vs. NT | **** | <0.0001 |
| HPBCD (H) vs. GT | ns | 0.4378 |
| HPBCD (H) vs. DT | **** | <0.0001 |
| HPBCD (H) vs. HM | ns | 0.9975 |
| HPBCD (H) vs. NM | **** | <0.0001 |
| HPBCD (H) vs. GM | ns | 0.1177 |
| HPBCD (H) vs. DM | **** | <0.0001 |
| NaCMC (N) vs. Hydrolyzed Gelatin (G) | **** | <0.0001 |
| NaCMC (N) vs. Dextran 20 kDa (D) | ns | 0.2767 |
| NaCMC (N) vs. Trehalose (T) | **** | <0.0001 |
| NaCMC (N) vs. Mannitol (M) | **** | <0.0001 |
| NaCMC (N) vs. HT | **** | <0.0001 |
| NaCMC (N) vs. NT | **** | <0.0001 |
| NaCMC (N) vs. GT | **** | <0.0001 |
| NaCMC (N) vs. DT | **** | <0.0001 |
| NaCMC (N) vs. HM | **** | <0.0001 |
| NaCMC (N) vs. NM | **** | <0.0001 |
| NaCMC (N) vs. GM | **** | <0.0001 |
| NaCMC (N) vs. DM | *** | 0.0001 |
| Hydrolyzed Gelatin (G) vs. Dextran 20 kDa (D) | **** | <0.0001 |
| Hydrolyzed Gelatin (G) vs. Trehalose (T) | ** | 0.0045 |
| Hydrolyzed Gelatin (G) vs. Mannitol (M) | **** | <0.0001 |
| Hydrolyzed Gelatin (G) vs. HT | ns | >0.9999 |
| Hydrolyzed Gelatin (G) vs. NT | **** | <0.0001 |
| Hydrolyzed Gelatin (G) vs. GT | ns | 0.8680 |
| Hydrolyzed Gelatin (G) vs. DT | **** | <0.0001 |
| Hydrolyzed Gelatin (G) vs. HM | ns | >0.9999 |
| Hydrolyzed Gelatin (G) vs. NM | **** | <0.0001 |
| Hydrolyzed Gelatin (G) vs. GM | ns | 0.4432 |
| Hydrolyzed Gelatin (G) vs. DM | **** | <0.0001 |
| Dextran 20 kDa (D) vs. Trehalose (T) | **** | <0.0001 |
| Dextran 20 kDa (D) vs. Mannitol (M) | ns | 0.0652 |
| Dextran 20 kDa (D) vs. HT | **** | <0.0001 |
| Dextran 20 kDa (D) vs. NT | ** | 0.0097 |
| Dextran 20 kDa (D) vs. GT | **** | <0.0001 |
| Dextran 20 kDa (D) vs. DT | **** | <0.0001 |
| Dextran 20 kDa (D) vs. HM | **** | <0.0001 |
| Dextran 20 kDa (D) vs. NM | ns | 0.3521 |
| Dextran 20 kDa (D) vs. GM | **** | <0.0001 |
| Dextran 20 kDa (D) vs. DM | ns | 0.4342 |
| Trehalose (T) vs. Mannitol (M) | **** | <0.0001 |
| Trehalose (T) vs. HT | ** | 0.0011 |
| Trehalose (T) vs. NT | **** | <0.0001 |
| Trehalose (T) vs. GT | **** | <0.0001 |
| Trehalose (T) vs. DT | * | 0.0394 |
| Trehalose (T) vs. HM | *** | 0.0009 |
| Trehalose (T) vs. NM | **** | <0.0001 |
| Trehalose (T) vs. GM | **** | <0.0001 |
| Trehalose (T) vs. DM | **** | <0.0001 |
| Mannitol (M) vs. HT | **** | <0.0001 |
| Mannitol (M) vs. NT | ns | >0.9999 |
| Mannitol (M) vs. GT | **** | <0.0001 |
| Mannitol (M) vs. DT | * | 0.0207 |
| Mannitol (M) vs. HM | **** | <0.0001 |
| Mannitol (M) vs. NM | ns | >0.9999 |
| Mannitol (M) vs. GM | **** | <0.0001 |
| Mannitol (M) vs. DM | ns | 0.9998 |
| HT vs. NT | **** | <0.0001 |
| HT vs. GT | ns | 0.9819 |
| HT vs. DT | **** | <0.0001 |
| HT vs. HM | ns | >0.9999 |
| HT vs. NM | **** | <0.0001 |
| HT vs. GM | ns | 0.7343 |
| HT vs. DM | **** | <0.0001 |
| NT vs. GT | **** | <0.0001 |
| NT vs. DT | ns | 0.1208 |
| NT vs. HM | **** | <0.0001 |
| NT vs. NM | ns | 0.9803 |
| NT vs. GM | **** | <0.0001 |
| NT vs. DM | ns | 0.9595 |
| GT vs. DT | **** | <0.0001 |
| GT vs. HM | ns | 0.9870 |
| GT vs. NM | **** | <0.0001 |
| GT vs. GM | ns | >0.9999 |
| GT vs. DM | **** | <0.0001 |
| DT vs. HM | **** | <0.0001 |
| DT vs. NM | ** | 0.0018 |
| DT vs. GM | **** | <0.0001 |
| DT vs. DM | ** | 0.0011 |
| HM vs. NM | **** | <0.0001 |
| HM vs. GM | ns | 0.7659 |
| HM vs. DM | **** | <0.0001 |
| NM vs. GM | **** | <0.0001 |
| NM vs. DM | ns | >0.9999 |
| GM vs. DM | **** | <0.0001 |

(**** stands for p<0.0001, *** for p<0.001, ** for p<0.01, * for p<0.05 and ‘ns’ for not significant difference).

**Table S2.** Differences in the X-ray diffractogram peak height for sodium chloride in the different spray-dried BSA-polymer formulations at T_0_ and T_90_ (See Figure 3 for changes in NaCl peaks)

| **#** | **Formulations** | **Peak intensity of NaCl peak observed at 2θ ~ 32°** | | |
| --- | --- | --- | --- | --- |
|  |  | **T_0_ (CPS)** | **T_90_ (CPS)** | **Delta (T_90_-T_0_)** |
| 0 | Control (C) | 2403 | 6128 | 3725 |
| 1 | HPβCD (H) | 3122 | 6833 | 3711 |
| 2 | NaCMC (N) | 1189 | 6037 | 4848 |
| 3 | Hydrolyzed Gelatin (G) | 2015 | 5031 | 3016 |
| 4 | Dextran 20k (D) | 1376 | 6129 | 4753 |
| 5 | Trehalose (T) | 1833 | 4316 | 2483 |
| 6 | Mannitol (M) | 2006 | 9570 | 7564 |
| 7 | HT | 1015 | 5116 | 4101 |
| 8 | NT | 1656 | 4938 | 3282 |
| 9 | GT | 1157 | 3754 | 2597 |
| 10 | DT | 1001 | 5892 | 4891 |
| 11 | HM | 1894 | 6237 | 4343 |
| 12 | NM | 1304 | 6313 | 5009 |
| 13 | GM | 1208 | 2758 | 1550 |
| 14 | DM | 1153 | 5748 | 4595 |

CPS: counts per second

**Table S3.** Powder density and flowability for selected spray-dried BSA formulations at T_0_

| **#** | **Formulations** | **Density (g/cm^3^)** | | **Flowability** | |
| --- | --- | --- | --- | --- | --- |
|  |  | **Bulk** | **Tapped** | **Carr**  **Index** | **Hausner Ratio** |
| 0 | Control (C) | 0.19 ± 0.02 | 0.24 ± 0.03 | 18.18 ± 1.45 | 1.22 ± 0.02 |
| 1 | HPβCD (H) | 0.18 ± 0.01 | 0.20 ± 0.02 | 13.44 ± 2.43 | 1.15 ± 0.03 |
| 3 | Hydrolyzed Gelatin (G) | 0.16 ± 0.01 | 0.21 ± 0.01 | 19.54 ± 1.91 | 1.24 ± 0.03 |

Bulk and tapped density measurements were performed using a 10 mL graduated cylinder. An appropriate amount (1-2 grams) of spray-dried powder was added to the cylinder, and the sample was subjected to 500 taps until no further reduction in powder volume was observed. The sample weight, bulk volume, and tapped volume were recorded, and the respective densities were calculated using the ratio of the sample mass to the measured volume. Flowability was assessed by calculating the Carr Index and Hausner Ratio.

As summarized in Table S3, the bulk and tapped densities of all three selected formulations were comparable, indicating similar packing behavior. However, the HPβCD-containing formulation exhibited slightly improved flowability compared to both the hydrolyzed gelatin and control formulations.


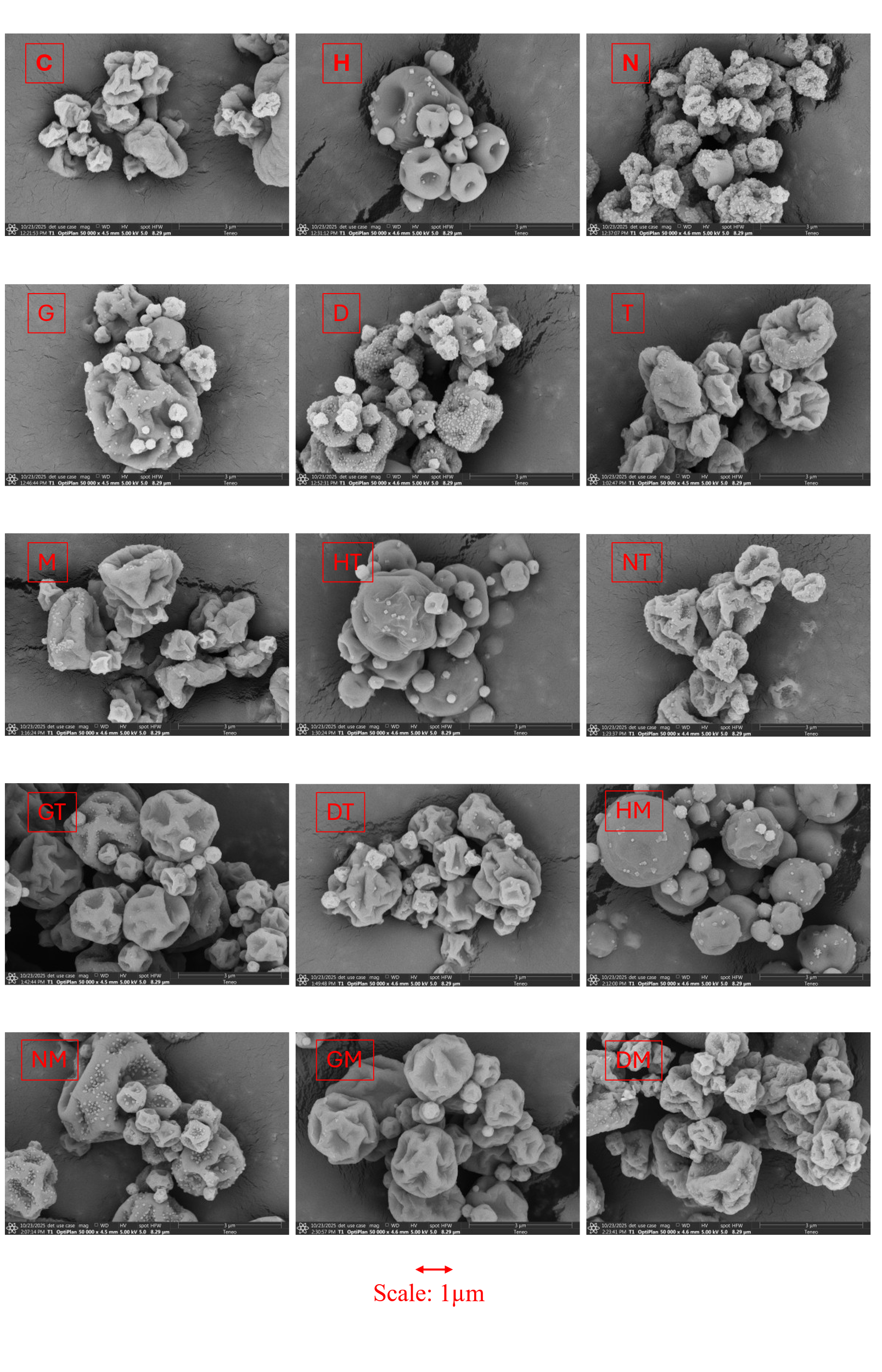


Figure S1. Morphology of spray-dried particles at T_90_.

Differential scanning calorimetry (DSC) was performed for select formulations at T_0_, including those containing HPβCD (A) and NaCMC (B). As shown in the representative thermograms below, no distinct Tg could be detected for these spray-dried powders, likely due to the presence of multiple amorphous components and potential overlap with protein and excipient relaxation events.


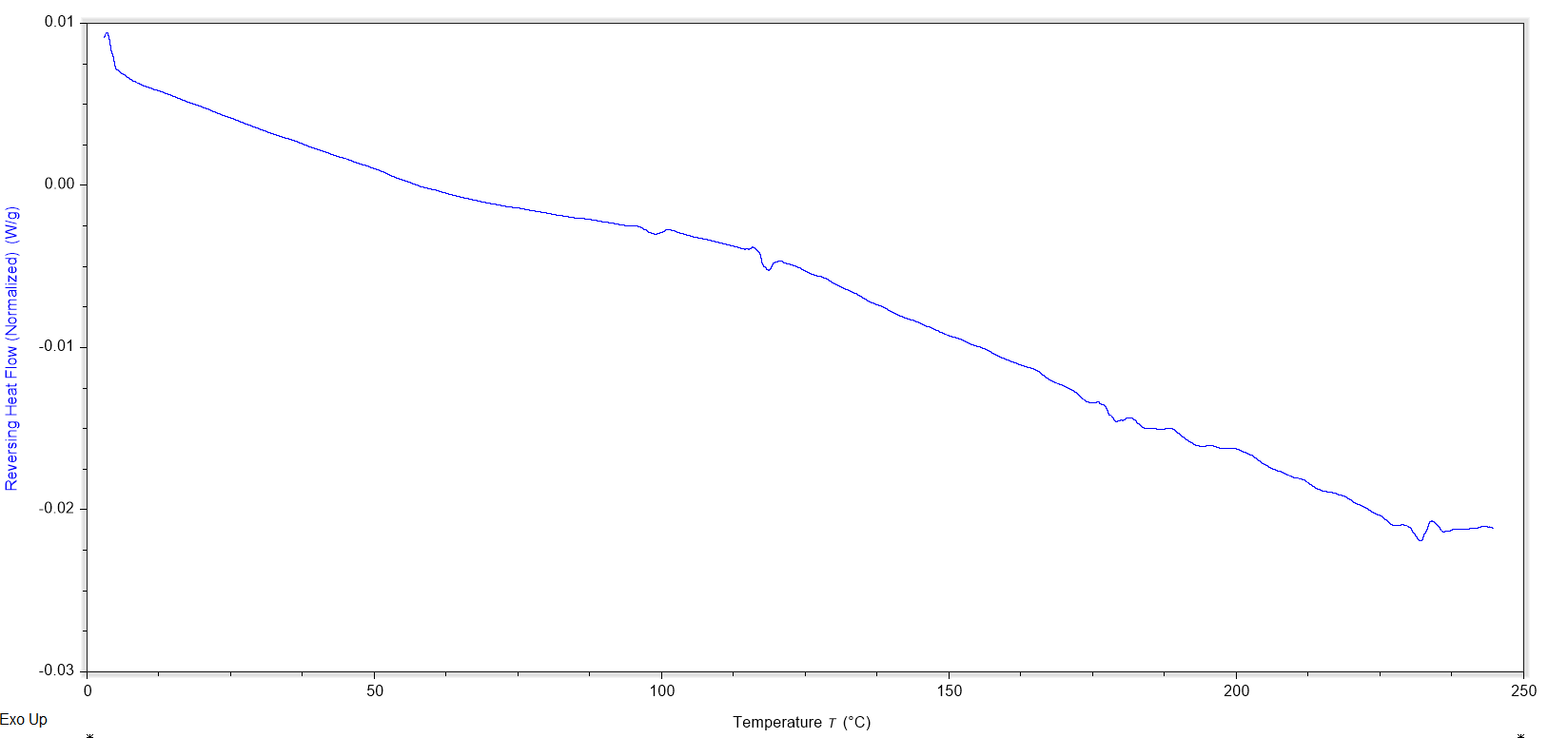


Figure S2A. DSC thermogram (A) of formulation 1 [containing BSA: HPβCD 1:1 w/w]


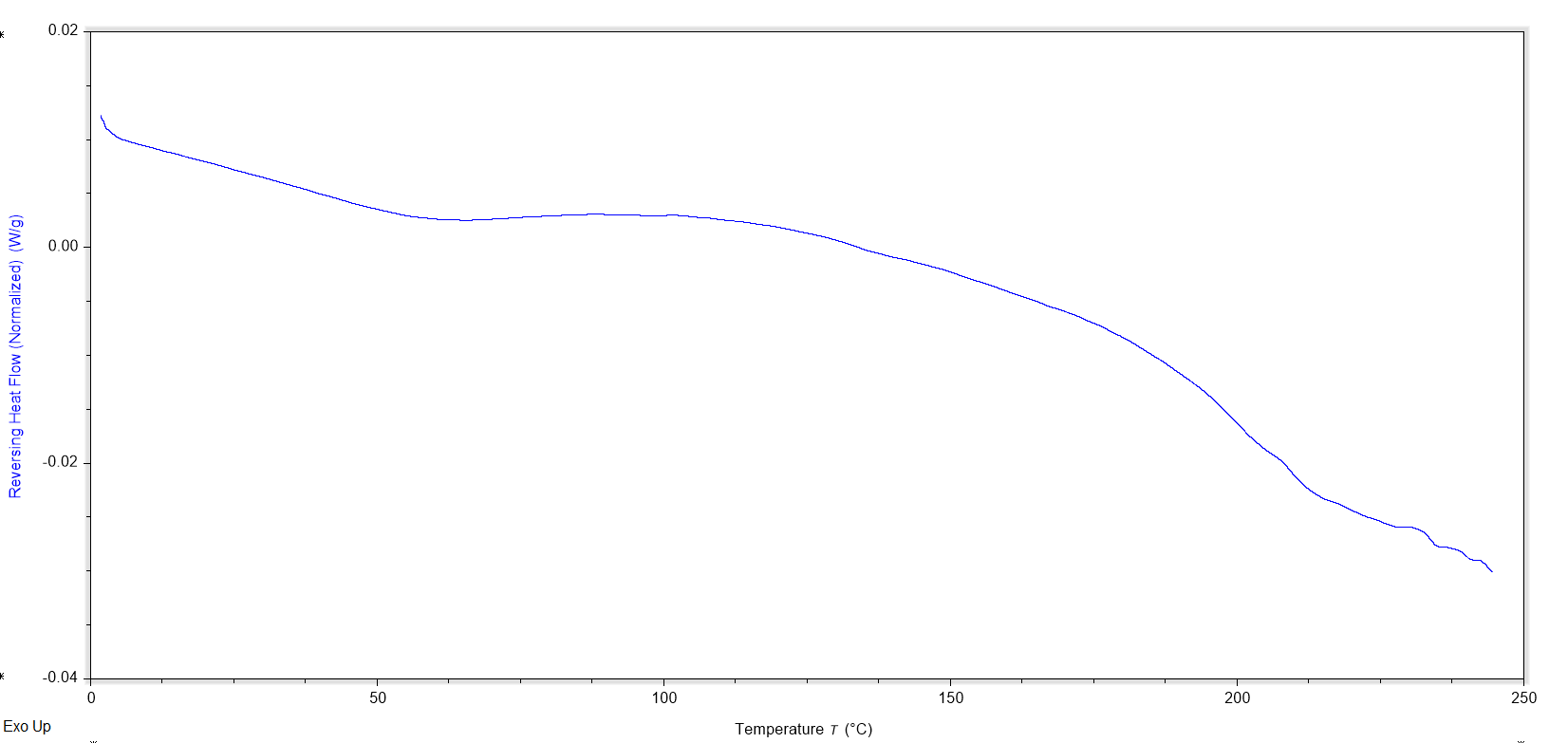


Figure S2B. DSC thermogram (B) of formulation 2 [containing BSA: NaCMC 1:1 w/w]
